# A rare-variant test for high-dimensional data

**DOI:** 10.1038/ejhg.2017.90

**Published:** 2017-05-24

**Authors:** Marika Kaakinen, Reedik Mägi, Krista Fischer, Jani Heikkinen, Marjo-Riitta Järvelin, Andrew P Morris, Inga Prokopenko

**Affiliations:** 1 Department of Genomics of Common Disease, Imperial College London, London, UK; 2 Estonian Genome Center, University of Tartu, Tartu, Estonia; 3 Neuroepidemiology and Ageing (NEA) Research Unit, Imperial College London, London, UK; 4 Department of Epidemiology and Biostatistics, MRC-PHE Centre for Environment and Health, School of Public Health, Imperial College London, London, UK; 5 Center for Life Course Health Research, University of Oulu, Oulu, Finland; 6 Unit of Primary Care, Oulu University Hospital, Oulu, Finland; 7 Biocenter Oulu, University of Oulu, Oulu, Finland; 8 Department of Biostatistics, University of Liverpool, Liverpool, UK

## Abstract

Genome-wide association studies have facilitated the discovery of thousands of loci for hundreds of phenotypes. However, the issue of missing heritability remains unsolved for most complex traits. Locus discovery could be enhanced with both improved power through multi-phenotype analysis (MPA) and use of a wider allele frequency range, including rare variants (RVs). MPA methods for single-variant association have been proposed, but given their low power for RVs, more efficient approaches are required. We propose multi-phenotype analysis of rare variants (MARV), a burden test-based method for RVs extended to the joint analysis of multiple phenotypes through a powerful reverse regression technique. Specifically, MARV models the proportion of RVs at which minor alleles are carried by individuals within a genomic region as a linear combination of multiple phenotypes, which can be both binary and continuous, and the method accommodates directly the genotyped and imputed data. The full model, including all phenotypes, is tested for association for discovery, and a more thorough dissection of the phenotype combinations for any set of RVs is also enabled. We show, via simulations, that the type I error rate is well controlled under various correlations between two continuous phenotypes, and that the method outperforms a univariate burden test in all considered scenarios. Application of MARV to 4876 individuals from the Northern Finland Birth Cohort 1966 for triglycerides, high- and low-density lipoprotein cholesterols highlights known loci with stronger signals of association than those observed in univariate RV analyses and suggests novel RV effects for these lipid traits.

## Introduction

In the past decade, thousands of loci for hundreds of phenotypes have been identified through genome-wide association studies (GWAS). Despite these discoveries, the issue of missing heritability^1^ remains unsolved for most complex traits. While real-life analyses and power estimations suggest improvements for discovery with very large sample sizes, for many complex traits the numbers required are unrealistic. A cost-effective alternative for improved power is to jointly analyse multiple traits, by taking advantage of the correlation structure between them.^2, 3, 4, 5, 6^

A number of methods for multi-phenotype analysis (MPA) have been developed, including dimension reduction, and multivariate and graphical models.^7^ In genetics, MPA was first proposed for linkage studies,^2, 3, 8, 9^ and recently, a plethora of methods for single-variant MPA of association has been suggested.^10^ MPA has been shown to provide a boost in power for locus discovery,^2, 3, 4, 5^ as well as increased precision of parameter estimates.^9^ There is also a biological advantage in analysing correlated traits jointly, since detecting loci that affect a combination of phenotypes could provide suggestions of pleiotropic effects,^7^ that is, one locus affecting multiple phenotypes in parallel. Indeed, many of the identified loci from single-phenotype analyses overlap, especially for epidemiologically correlated traits, for example, glycaemic traits share up to a half of the loci with other cardiometabolic traits, including type 2 diabetes, lipids, measures of central obesity, height, blood pressure and hypertension.^11^ This overlap suggests shared genetic architecture underlying at least a part of the observed epidemiological correlations. With joint analysis of multiple correlated phenotypes, the potentially shared genetic architecture would be better addressed by direct joint modelling. In addition, current technology allows for cost-effective deep phenotyping^12^ such as the production of the metabolomics data including hundreds or even thousands of variables. These large-scale omics datasets will be necessary in understanding the exact relationship between genes and phenotypes. However, the great potential held by the omics data will only be achieved via integration and development of highly scalable computational and quantitative approaches.^13^ MPA methods will be particularly useful for the omics data in order to reduce dimensionality and avoid the penalties posed by correction for multiple testing.

Another way to improve power for locus discovery is to allow for a wider allele frequency range, including low-frequency and rare variants (minor allele frequency, MAF<5%, both denoted here by RVs). Development in technology to produce high-quality, low-cost sequencing data has had many positive implications for RV identification. Although whole-genome/-exome sequencing is still not feasible at a large scale, that is, sequencing hundreds of thousands of individuals, accurate RV data can be produced with imputation based on dense reference panels such as the 1000 genomes project,^14^ the UK10K Project^15^ or the Haplotype Reference Consortium.^16^ For example, imputation to the UK10K reference panel including 7562 haplotypes, after re-phasing with SHAPEIT v2,^17^ yielded for variants with MAF as low as 0.1% an *r*^*2*^=0.5 with genotyped variants.^18^ Combining these factors has led to an increased interest in RV association analysis, resulting in the development of burden tests using collapsing techniques, variance-component tests and combinations of these two.^19^

**Figure 1:**
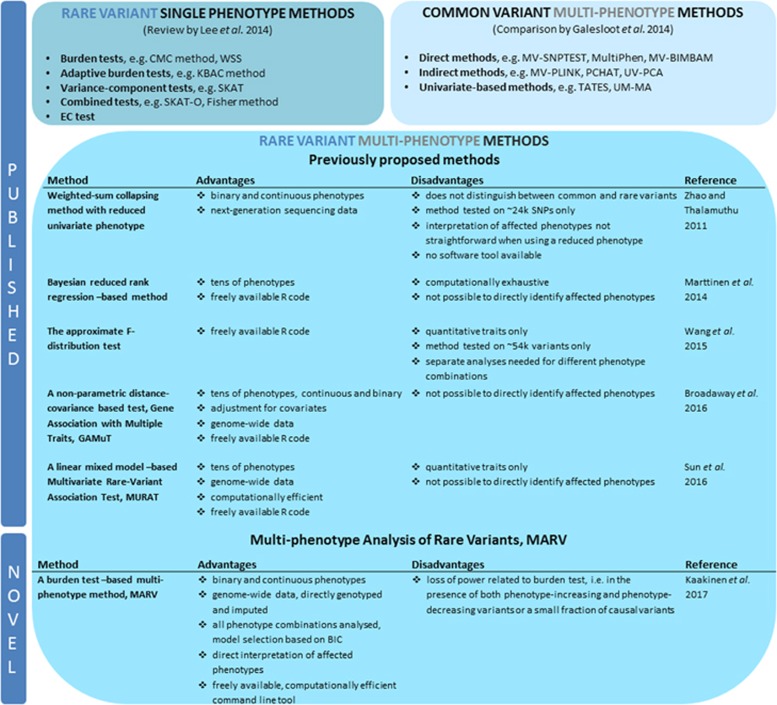
Comparison of MARV with previously proposed RV multi-phenotype association analysis methods. Upper blocks: Established rare-variant single-phenotype methods^19^ and common-variant multi-phenotype methods^10^ based on the individual level data. Lower block: Previously proposed RV multiple-phenotype methods^20, 21, 22, 23, 24^ versus our proposed MARV method.

We suggest integrating MPA with RV association analysis to further increase the power of locus discovery and to provide novel biological insights into complex trait genetics. Currently available methods for MPA are mostly tailored for single-variant associations, and are thus underpowered to detect RV associations. Some recently developed methods have addressed MPA for RVs,^20, 21, 22, 23, 24^ but they suffer, for example, from scalability limitations, their inability to combine continuous and binary phenotypes or to analyse different phenotype combinations, and finally, the lack of efficient computational tools (Figure 1). Here we introduce a burden test-based multi-phenotype analysis of RVs, MARV, which has several advantages over the few previously proposed methods that we are aware of: it is applicable to sequencing, imputed or genotyping data, allows for both binary and continuous phenotypes, provides association results for all phenotype combinations, including univariate tests within one run, and is implemented into a simple to use and computationally efficient software tool that can analyse millions of variants (Figure 1).

## Materials and methods

### Multi-phenotype analysis of rare variants through reverse regression

Our method is based on the RV burden test approach,^25, 26^ in which RVs within a genomic region, defined by positional boundaries and/or annotation, for example, are collapsed into one variable. Specifically, within a genomic region, we calculate the proportion of RVs at which an individual *i* carries minor alleles: *r*_*i*_*n*_*i*_^−1^, where *r*_*i*_ is the number of minor alleles at RVs and *n*_*i*_ is the total number of RVs. We then model this proportion as a linear combination of *K* phenotypes, that is, we use ‘reverse regression’ as compared to standard GWAS, with the genotype data as the outcome and the phenotypes as the predictors, in the same way as in the MultiPhen approach for common variants.^27^ Thus, the model becomes


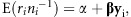


where *r*_*i*_*n*_*i*_^−1^ is the proportion of minor alleles for *i*th individual, **y**_i_ represents the phenotype data for individual *i*, with corresponding regression coefficients **β**=(*β*_1_,…, *β*_*K*)_. The modelling is performed via weighted linear regression with the following weights: the proportion of successfully genotyped or imputed RVs within the region of interest. Parameter estimates are obtained via least squares estimation. Then, a likelihood ratio test is constructed by comparing the weighted log likelihoods of the fitted model against a null model where **β**=0. The test statistic has an approximate *χ*^2^ distribution with *K* degrees of freedom. The method described has been implemented in a freely available software tool MARV,^28^ which is available at https://github.com/ImperialStatGen/MARV.

The methodology is flexible such that it can accommodate both quantitative and binary phenotypes, as well as directly genotyped and imputed RVs. For imputed RVs, instead of using the 0/1 indicator for the absence/presence of minor allele, we use the posterior probability that an individual is heterozygous or rare homozygous. For discovery purposes, the full model, including all the phenotypes is fitted. However, to allow further investigation of the loci reaching genome-wide significance after correction for multiple testing to take into account the number of regions tested within the analysis, we have implemented in the MARV software the possibility to analyse all phenotype combinations. For model selection purposes, MARV further calculates the Bayesian information criterion, BIC*=*−2•ln(*L*)*+ K*•ln(*N*), where *L* is the estimated likelihood of the model, *K* is the number of parameters and *N* is the number of observations.

### Genotype simulations

To evaluate the type I error rate and power of our method, we simulated genetic variants by using a model that results in an allele spectrum similar to that observed in the European population.^29^ Specifically, we used the forward simulation software tool ForSim^30^ and the hybrid model for population history proposed previously.^29^ In brief, the ancestral population size was assumed to be 8100 for 50 000 generations, followed by a bottleneck population size of 2000. After this time the population expands with an exponential growth rate of 1.29% for over 370 generations, resulting in a modern effective population size of 227 650. With a maximum number of offspring set to two, the simulation resulted in a population of about 500 000 individuals. The mutation rate was assumed to be 2.0 × 10^−8^ bp per generation. This hybrid model has features of two published demographic histories^31, 32^ and has been shown to recapitulate the number and frequency distributions of both rare and common synonymous sites, as well as empirically observed patterns of linkage disequilibrium between common variants.^29^

We modelled loci as a series of 8 exons and 7 introns (alternating), with exons of length 300 bp and introns of length 3 kb such that the total coding length is 2.4 kb and the total transcript length is 23.4 kb, which corresponds to an “average” protein-coding gene from the RefSeq database.^29^ Around each transcript, we also simulated 100 kb of neutral genomic target flanking both sides of the gene.^29^ The simulation resulted in 16 616 SNPs, of which 96% have a MAF<5%. We randomly sampled SNPs as causal irrespective of their functional impact such that the MAF of any individual causal SNP was <2% and the sum of the MAFs did not exceed 5%.^33^ This resulted in 146 causal SNPs that were used for all the tested scenarios. For the selected SNPs, we randomly sampled individuals from this final population to achieve 10 000 replicates of studies of 1000 and 5000 individuals.

### Phenotype simulations

We simulated two continuous phenotypes from a multivariate normal distribution, such that


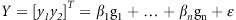


where g_k_ is the causal variant and *β*_*k*_ the effect for each causal variant, *k*=1,…,146, and *ε* ~ MVN(0,Σ_*Y*_), Σ_*Y*_=cov(*y*_1_,*y*_2_) =
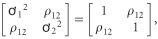
, and 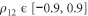 ϵ [−0.9, 0.9]. We used the function mvrnorm in the statistical software R.^34^ The genetic effects *β*_*k*_ were considered under three scenarios, including (1) no effect (for type I error rate assessment), that is, *β*_*k*_=0; (2) all causal genetic variants having a trait-increasing effect (*β*_*k*_=0.1), and (3) half of the causal variants having a trait-increasing effect (*β*_*k*_=0.1) and half having a trait-decreasing effect (*β*_*k*_=−0.1). Scenarios (2) and (3) were further considered *in situations* when there are effects on (a) both phenotypes in the same direction with same magnitudes, (b) both phenotypes in opposing directions with same magnitudes, (c) both phenotypes in the same direction with different magnitudes (the effect on phenotype *two* is half of that for phenotype *one*), and d) only on one of the phenotypes.

### Type I error rate and power estimation

We estimated the type I error rate and power of our method using the full model by calculating the proportion of the 10 000 replicate analyses, in which *P*<0.05 for the test of association. Since we analysed only the full model and one genomic region, no correction for multiple testing was applied.

We compared the power of MARV to that of a recently published multi-phenotype RV method GAMuT,^23^ which is based on a non-parametric distance-covariance test. This method requires a phenotype similarity matrix, either by using a projection matrix or by using user-selected linear kernel functions. For our estimates, we constructed the similarity projection matrix. The genotypic similarity matrix was calculated assuming a linear kernel for genotypes, where weights are functions of MAF, following the notation from Broadaway *et al.*^23^ The method then constructs the test and provides the *P*-value as the output. Similarly to MARV, we estimated the power of GAMuT by calculating the proportion of the 10 000 replicate analyses in which *P*<0.05 for the test of association.

In addition, we compared the power of MARV over a univariate RV test by calculating associations between the simulated RVs and the two phenotypes separately using the GRANVIL software.^26^ To assess power, we calculated the proportion of tests for which *P*<0.025, where the level of significance is Bonferroni corrected for two tests performed.

### Experimental setup

To examine our method with a real dataset, we analysed the data for triglycerides (TG), low-density lipoprotein (LDL) and high-density lipoprotein (HDL) cholesterol from individuals belonging to the Northern Finland Birth Cohort 1966 (NFBC1966) and having participated in the 31-year clinical examination. The cohort covers over 96% of all births in the two northernmost provinces of Finland in 1966 (*N*=12 068 live-born children).^35^ The Ethics Committees of the University of Oulu and Northern Ostrobothnia Hospital District have approved the study, and the individuals used for the analyses have provided written informed consent.

At the clinical examination, blood samples were drawn after overnight fasting. Samples were stored at –70 °C until analysed. Enzymatic assays of fasting HDL cholesterol and TG were measured using Hitachi 911 Clinical Chemistry Analyzer and commercial reagents (Boehringer, Mannheim, Germany) in the accredited laboratory of Oulu University Hospital. LDL was obtained using a previously described method.^36^ DNA was extracted and genome-wide genotyping was performed with the Illumina HumanCNV370DUO Analysis BeadChip platform at the Broad Institute, USA. The Beadstudio algorithm was used for calling the genotypes. Detailed genotyping and sample quality control (QC) of the first set of data have been reported previously.^37^ More samples were genotyped afterwards, and after the genotyping data QC, there were 5402 subjects and 324 896 SNPs available for analysis. SNPs were imputed to the 1000 Genomes all ancestries reference panel (March 2012) with IMPUTE2,^38^ resulting in ~38M SNPs for analysis. Of these, 7 396 876 markers were non-monomorphic with MAF<5% and good imputation quality (info>0.4).

For the phenotypes, we excluded non-fasting individuals and those known to be on lipid-lowering medication. Residuals of TG, LDL and HDL were calculated by adjusting for sex, body mass index and the first three principal components (PCs) derived from the genetic data to control for potential population structure. The residuals were further inverse-normal transformed; however, it is worth noticing that a transformation to reduce skewness is not a prerequisite for the software as the phenotypes are not the outcomes of interest here. GWAS and the phenotype data were available for 4876 individuals.

The multi-phenotype genome-wide RV analysis using the transformed residuals was performed with MARV using the method “expected”, that is, we used the genotype dosages coming from imputation. To define gene regions across the genome, we used the gene list from the University of California Santa Cruz (UCSC, NCBI genome sequence build 37, hg19).^39^ We applied a Bonferroni correction for 30 000 genes resulting in a level of significance of 1.67 × 10^−6^. We analysed all variants irrespective of their annotation across autosomal chromosomes using the following thresholds: MAF<5% and imputation quality>0.4.

## Results

### Type I error rate and power

**Figure 2:**
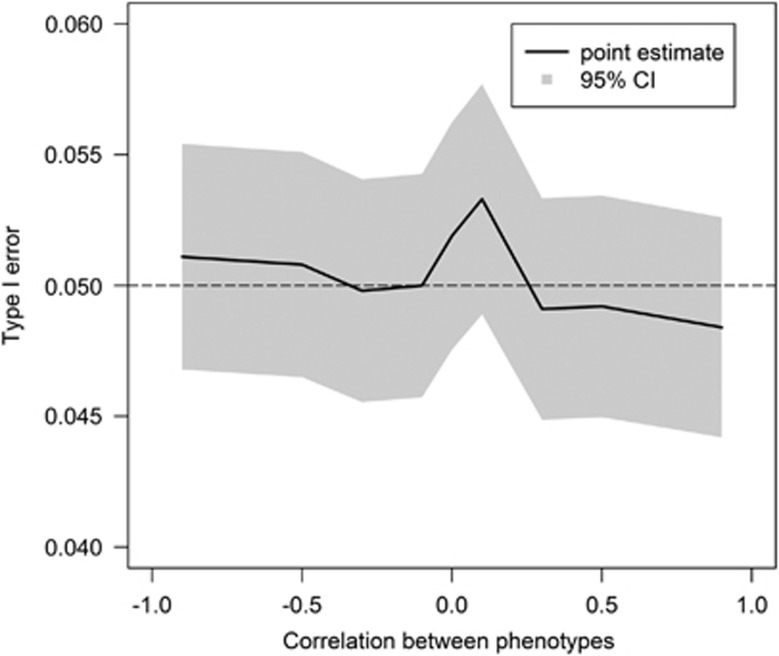
Estimated type I error rate with 95 % confidence interval (CI) of the MARV method with *N*=5000 and varying correlation between two continuous phenotypes. The following correlations were evaluated: −0.9, −0.5, −0.3, −0.1, 0, 0.1, 0.3, 0.5, 0.9.

The simulation studies on 5000 individuals showed a good control of type I error rate under various correlation structures between the two continuous phenotypes (Figure 2). Supplementary Figure S1 demonstrates that the control is maintained with a substantially smaller sample size (*N*=1000). Numerical results for both sample sizes are provided in Supplementary Table S1.

**Figure 3:**
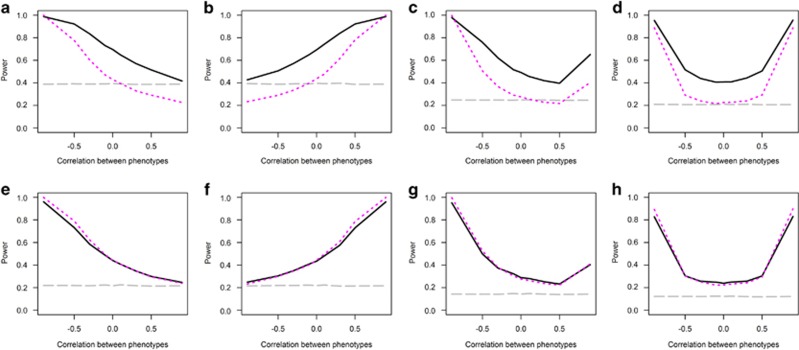
Statistical power of the MARV method with *N*=5000 and varying correlation between two continuous phenotypes. (**a**–**d**) All genetic effects are trait-increasing. (**e**–**h**) Half of the genetic effects are trait-increasing, half trait-decreasing. (**a**,**e**) Effects on both phenotypes, same direction, same magnitude. (**b**,**f**) Effects on both phenotypes, opposite direction, same magnitude. (**c**,**g**) Effects on both phenotypes, same direction, different magnitude (effect on phenotype 2 is half of that on phenotype 1). (**d**,**h**) Effects on one phenotype only. Solid, black line: MARV; dotted, magenta line: GAMuT; dashed, grey line: univariate analysis (GRANVIL). The following correlations were evaluated: −0.9, −0.5, −0.3, −0.1, 0, 0.1, 0.3, 0.5, 0.9.

In all the tested scenarios, when all genetic effects are trait-increasing, power is almost always higher than that of univariate analyses or the kernel-based multi-phenotype test GAMuT (Figures 3a–d and Supplementary Table S2). When the genetic effects are in the same direction and of the same magnitude for the two phenotypes (Figure 3a), power is highest when the correlation between the phenotypes is highly negative. The opposite is observed, that is, power is highest with high positive correlation between the phenotypes when the genetic effects are in the opposite directions but of the same magnitude (Figure 3b). When there are genetic effects in the same direction, but with different magnitude (half of the effect size of phenotype 1 in phenotype 2), the pattern of power follows that of the scenario in Figure 3a, that is, power is higher with negative correlation between the phenotypes (Figure 3c). However, the power decreases at a faster rate compared to 3a) and increases again when the correlation is equal to or higher than 0.5. Finally, when the genetic variants affect only one of the correlated phenotypes, power is increased both when the phenotypes are highly positively or highly negatively correlated (Figure 3d). Even with no correlation between the phenotypes, there is an improvement in power over univariate analyses, which is due to the avoidance of correction for multiple testing with our multivariate model, despite the requirement of an additional degree of freedom. Results from simulations using a sample size of 1000 individuals are shown in Supplementary Figure S2. Even with a smaller sample size, joint analysis of correlated phenotypes offers an improvement in power over the univariate analysis of 5000 individuals when the correlation between the phenotypes is high enough (over 0.5 or less than −0.5).

When we considered the above mentioned four scenarios (Figures 3a–d) under the assumption that half of the genetic variants are trait-increasing and half trait-decreasing, we see similar patterns of power as for the situation of all variants being trait-increasing, but in general, the power is lower (Figures 3e–h and Supplementary Table S2). In all cases, MARV performed better than univariate analyses. The power of GAMuT was only very modestly increased over that of MARV with both, trait-increasing and -decreasing directions of genetic effects on highly correlated phenotypes.

### Real data example

**Figure 4:**
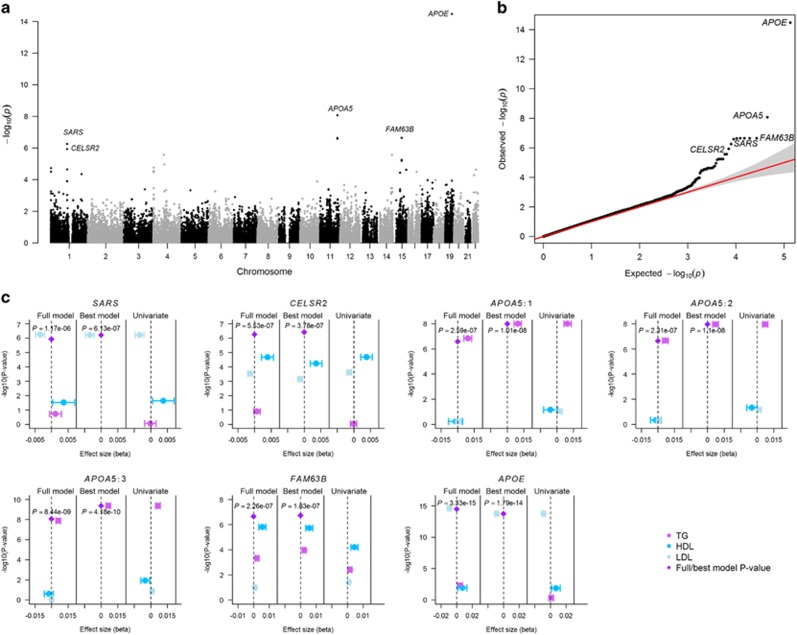
Genome-wide association analysis results from MARV for triglycerides, high-density lipoprotein and low-density lipoprotein cholesterols in the NFBC1966. (**a**) Manhattan plot for the full model statistical significance. Genes reaching statistical significance (*P*<1.67 × 10^−6^) are annotated. (**b**) QQ-plot of the full model association *P*-values against the expected *P*-values. Note that at some of the loci, different gene transcripts resulted in exactly the same association result. Such results show as a horizontal line of dots in the figure. (**c**) Effect sizes with their 95% confidence intervals of triglycerides, high-density lipoprotein and low-density lipoprotein cholesterols plotted against their statistical significance for the loci reaching genome-wide significance. In each figure, the panel on the left shows the results from the full model, the middle panel shows them from the best model based on Bayesian Information Criterion and the right panel illustrates results from univariate models. For *APOA5*, statistically significant associations were detected for three different transcripts.

The three selected phenotypes, TG, HDL and LDL, were modestly correlated with each other (R_TG_HDL_=−0.32, R_TG_LDL_=0.31, R_HDL_LDL_=−0.19). The MPA of RVs for the three phenotypes revealed genome-wide significant associations (*P*<1.67 × 10^−6^, Methods) covering five gene regions: *SARS*, *CELSR2*, *APOA5*, *FAM63B* and *APOE* (Figures 4a and b; Supplementary Tables S3 and S4). Besides the full model, MARV also provides parameter estimates and tests of associations for each phenotype combination, including the single-phenotype models. Therefore, we were able to compare the results from the joint analysis against traditional single-phenotype analysis. Additionally, the BIC provided by MARV for each sub model served for selection of the phenotype combination providing the best fit. At *SARS*, *APOA5* and *APOE*, the univariate models provided the best fit (Figure 4c; Supplementary Table S3). At *CELSR2* and *FAM63B*, the best fit was coming from the combination of LDL+HDL and TG+HDL, respectively, and only the full and best models reached genome-wide significance, indicating that the associations would have been missed in univariate analyses. Both of these loci are also highly enriched for RVs, with 87 variants with MAF<5% being included for *CELSR2* analysis and 284 for *FAM63B*, while for the other associated genes the rare-variant count ranged between 3 and 19 (Supplementary Tables S3 and S4).

## Discussion

We propose a novel flexible method, MARV, for MPA of RVs across the genome applicable to the sequencing, imputed and genotyping data in unrelated individuals. This method using the powerful reverse regression approach should enable novel discoveries based on the large-scale genomic data with high DNA-variant density. We show with simulations that our method has a good control of type I error rate under various scenarios, even with a sample size as small as 1000 individuals. The power of our method always exceeds that of a univariate RV burden test under the tested scenarios, and the patterns mirror those presented for a common-variant multi-phenotype method.^27^ Compared to a kernel-based multi-phenotype RV test, the power of MARV is notably increased when all the genetic effects are in the same direction, and is similar when half of the effects are protective and half are deleterious. The gain in power is observed due to the inclusion of correlated phenotypes in one model and thus, correction for multiple testing is avoided, given joint phenotype modelling. This feature is important for the efficient analysis of the large-scale high-dimensional omics data. For the univariate analyses, we applied the commonly used Bonferroni correction to account for two tests (one for each phenotype) performed. This notably decreased the power of the univariate method. We note that the Bonferroni correction applied may have been too stringent, especially when the phenotypes are highly correlated, but this approach is standard in GWAS^40^ and is widely applied to such traits.^41^

With application of MARV to TG, HDL and LDL phenotypes, we were able to detect genome-wide significant associations with RVs even with a sample size as modest as 4876 individuals. We found evidence for novel RV associations at *CELSR2*, *SARS* and *FAM63B,* of which common variation at *CELSR2* has been previously associated with blood lipids,^42^ and at *SARS/CELSR2* with type 2 diabetes,^43^ while *FAM63B* has no reported links with blood lipid levels. We identified two additional well-established lipid genes, *APOA5* and *APOE*.^42^ A recent study reported RV associations at *APOE* with lipid levels,^44^ and another study associated RVs at *APOA5* with myocardial infarction.^45^ Our findings are in line with previous reports of variation at *APOA5* and *APOE* mainly affecting TG and LDL levels, respectively;^42^ however, we observed RV association at *CELSR2* with both HDL and LDL cholesterol levels, thus extending previous reports on common variation at this locus about primary effects on LDL.^42^ The novel *FAM63B* has previously been associated with schizophrenia through methylation patterns,^46^ making this locus an interesting biological candidate for lipid metabolism and worth further investigation, as metabolic disturbances often co-occur with psychotic illnesses.^47^ Importantly, our analyses showed that the associations at this locus and at *CELSR2* would not have been observed in univariate analyses, whereas RV MPA had sufficient power to detect these signals. We also observed that MARV was able to detect effects from genes with widely varying sizes and number of RVs (*APOA5* with 2.5 Mbp and 3 RVs vs *FAM63B* with 86 Mbp and 284 RVs). It remains of future work, however, to formally assess the power of MARV under properties such as varying sets of causal variants, different gene size, mutation rate, haplotype length or degree of linkage disequilibrium between causal variants.

The simulated scenarios presented here are for two continuous phenotypes, but the method is easily applicable to a large number of phenotypes, as the real data example showed, and to a mixture of binary and continuous phenotypes. This is due to the methodological framework used, for example, reverse regression, in which the phenotypes are treated as predictors instead of outcomes, allowing for an easy incorporation of variables with varying properties into the model, as in any other linear regression analysis. Further, we have presented an example using a gene region-based association analyses, but the method and software are flexible and allow for analyses of any regions defined by the user based on start and end positions, for example, region sets, including those encoding active enhancers from analyses of human regulome.^48^ MARV could also be adapted to focus on only the most deleterious variants, rather than all of those mapping to a gene region via extracting or excluding specific markers.

There are limitations to our method and the current version of the software that warrant discussion. MARV is based on a burden test, which has been shown to be a powerful method for RV association testing when a large proportion of variants are causal and their effects are in the same direction.^19^ However, in the presence of both trait-increasing and trait-decreasing effects, or of only a small proportion of causal variants amongst those tested, there is a loss in power.^19^ As MARV is a burden test, it also suffers from some loss of power when both trait-increasing and trait-decreasing effects impact phenotypes, as shown in Figure 3. However, the power is still always higher compared to the univariate burden test. Similarly, we expect that power could be reduced in the presence of only a small number of causal variants.^49^ Combined burden and variance-component tests, such as SKAT-O, are claimed to be more robust than burden tests in the above mentioned scenarios.^19^ However, when we compared the power of MARV to a kernel-based multi-phenotype RV test GAMuT in the presence of both trait-increasing and trait-decreasing effects, the power of MARV is similar to that of GAMuT in all tested scenarios.

In summary, we have developed a powerful method to facilitate the discovery of RV genetic effects within the multi-phenotype framework. The method has been implemented into a freely available, easy to use software tool MARV, and should allow wide and rapid implementation in the analytical pipelines for large-scale high-dimensional datasets, therefore paving the way for novel genomic discoveries.
