# Supplementary material for: A rare-variant test for high-dimensional data
Source: Eur J Hum Genet. 2017 May 24;25(8):988–94. doi: 10.1038/ejhg.2017.90 (PMC5513099; doi:10.1038/ejhg.2017.90)
Supplement: Supplementary Figures and Tables [file ejhg201790x1.docx]

**
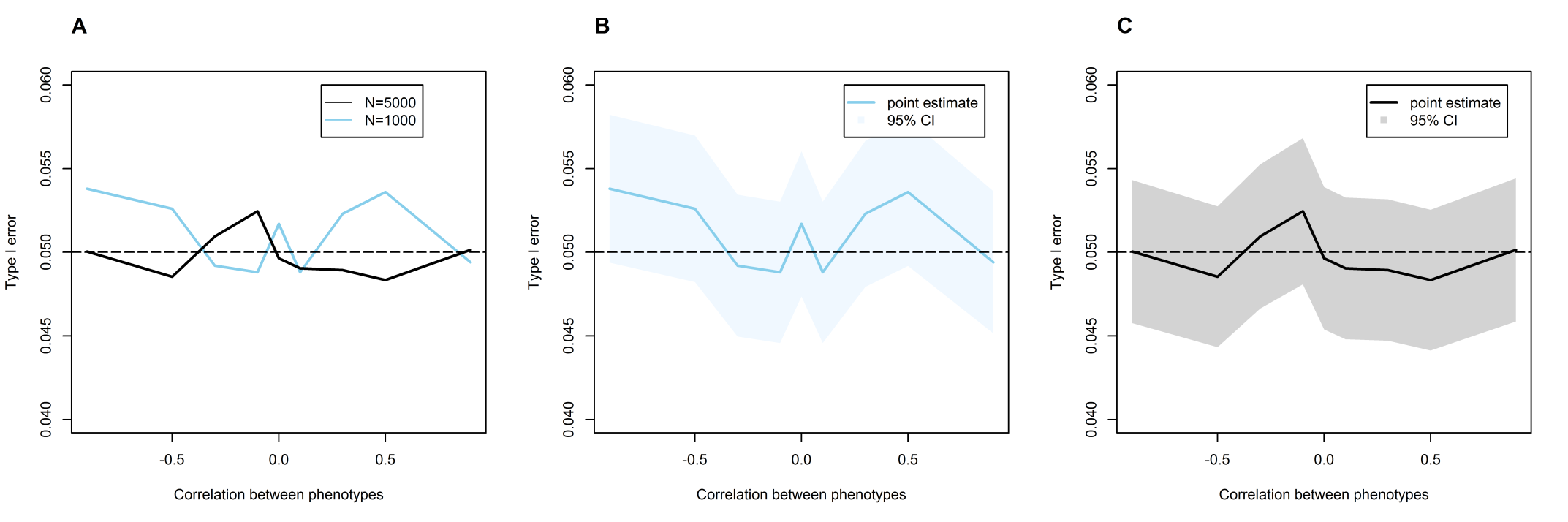
Figure S1.** **Type I error rate of the multi-phenotype analysis of rare variants (MARV) method with N=1,000 and N=5,000 and varying correlation between two continuous phenotypes.** A) Point estimates of the type I error rate estimates with N=1,000 and N=5,000; B) Point estimates and 95% confidence intervals (CI) of the type I error rate with N=1,000; B) Point estimates and 95% confidence intervals (CI) of the type I error rate with N=5,000. The following correlations were evaluated: -0.9, -0.5, -0.3, -0.1, 0, 0.1, 0.3, 0.5, 0.9.

**
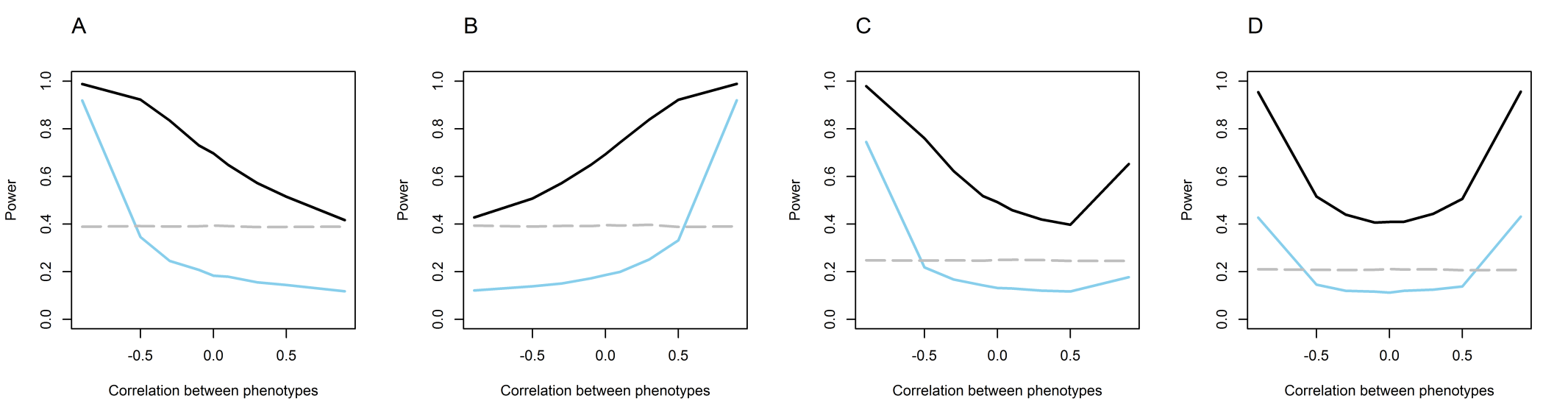
**

**Figure S2**. **Statistical power of the multi-phenotype analysis of rare variants (MARV) method by sample size and varying correlation between two continuous phenotypes.** All genetic effects are trait-increasing. A) Effects on both phenotypes, same direction, same magnitude, B) Effects on both phenotypes, opposite direction, same magnitude, C) Effects on both phenotypes, same direction, different magnitude (effect on phenotype 2 is half of that on phenotype 1), D) Effects on one phenotype only. Black line, MARV with N=5,000; blue line, MARV with N=1,000; grey line, univariate with N=5,000. The following correlations were evaluated: -0.9, -0.5, -0.3, -0.1, 0, 0.1, 0.3, 0.5, 0.9.

**Table S1.** **Type I error rate of MARV using the full model assessed by simulation.**

|  | **Correlation between traits** | | | | | | | | |
| --- | --- | --- | --- | --- | --- | --- | --- | --- | --- |
| **Sample size** | **-0.9** | **-0.5** | **-0.3** | **-0.1** | **0** | **0.1** | **0.3** | **0.5** | **0.9** |
| N=1,000 | 0.0538 | 0.0526 | 0.0492 | 0.0488 | 0.0517 | 0.0488 | 0.0523 | 0.0536 | 0.0494 |
| N=5,000 | 0.0500 | 0.0485 | 0.0509 | 0.0524 | 0.0496 | 0.0490 | 0.0489 | 0.0483 | 0.0501 |

**Table S2. Statistical power of MARV assessed by simulation.** To illustrate the increase in power by sample size, power with both N=1,000 and N=5,000 were calculated for all scenarios a-d in 1. For scenarios g-h in 2 only N=5,000 was considered.

|  | **Correlation between traits** | | | | | | | | |
| --- | --- | --- | --- | --- | --- | --- | --- | --- | --- |
|  | **-0.9** | **-0.5** | **-0.3** | **-0.1** | **0** | **0.1** | **0.3** | **0.5** | **0.9** |
| **1: All genetic effects are trait-increasing** | | | | | | | | | |
| *a. Effects on both phenotypes, same direction, same magnitude* | | | | | | | | | |
| N=1,000 | 0.9188 | 0.3458 | 0.2455 | 0.2077 | 0.1829 | 0.1790 | 0.1551 | 0.1439 | 0.1180 |
| N=5,000 | 0.9883 | 0.9226 | 0.8350 | 0.7304 | 0.6967 | 0.6487 | 0.5719 | 0.5150 | 0.4166 |
|  |  |  |  |  |  |  |  |  |  |
| *b. Effects on both phenotypes, opposite direction, same magnitude* | | | | | | | | | |
| N=1,000 | 0.1211 | 0.1389 | 0.1505 | 0.1721 | 0.1862 | 0.1991 | 0.2518 | 0.3315 | 0.9194 |
| N=5,000 | 0.4276 | 0.5071 | 0.5720 | 0.6486 | 0.6934 | 0.7425 | 0.8390 | 0.9217 | 0.9885 |
|  |  |  |  |  |  |  |  |  |  |
| *c. Effects on both phenotypes, same direction, different magnitude* | | | | | | | | | |
| N=1,000 | 0.7450 | 0.2182 | 0.1675 | 0.1430 | 0.1314 | 0.1298 | 0.1206 | 0.1173 | 0.1769 |
| N=5,000 | 0.9787 | 0.7598 | 0.6222 | 0.5178 | 0.4919 | 0.4582 | 0.4197 | 0.3972 | 0.6522 |
|  |  |  |  |  |  |  |  |  |  |
| *d. Effects on one phenotype only* | | | | | | | | | |
| N=1,000 | 0.427 | 0.1457 | 0.1197 | 0.1166 | 0.1121 | 0.1199 | 0.1244 | 0.1382 | 0.4311 |
| N=5,000 | 0.954 | 0.5159 | 0.4394 | 0.4062 | 0.4087 | 0.4096 | 0.4432 | 0.5055 | 0.9562 |
|  |  |  |  |  |  |  |  |  |  |
| **2: Half of the genetic effects are trait-increasing, half trait-decreasing (N=5,000)** | | | | | | | | | |
| *e. Effects on both phenotypes, same direction, same magnitude* | | | | | | | | | |
|  | 0.9623 | 0.7321 | 0.5852 | 0.4875 | 0.4410 | 0.4078 | 0.3479 | 0.2997 | 0.2466 |
|  |  |  |  |  |  |  |  |  |  |
| *f. Effects on both phenotypes, opposite direction, same magnitude* | | | | | | | | | |
|  | 0.2481 | 0.3046 | 0.3500 | 0.4078 | 0.4351 | 0.4818 | 0.5756 | 0.7296 | 0.9612 |
|  |  |  |  |  |  |  |  |  |  |
| *g. Effects on both phenotypes, same direction, different magnitude* | | | | | | | | | |
|  | 0.9526 | 0.4983 | 0.3735 | 0.3233 | 0.2889 | 0.2820 | 0.2522 | 0.2322 | 0.4036 |
|  |  |  |  |  |  |  |  |  |  |
| *h. Effects on one phenotype only* | | | | | | | | | |
|  | 0.8280 | 0.3041 | 0.2570 | 0.2486 | 0.2388 | 0.2498 | 0.2572 | 0.3034 | 0.8307 |
